# Brain Activity in Fairness Consideration during Asset Distribution: Does the Initial Ownership Play a Role?

**DOI:** 10.1371/journal.pone.0039627

**Published:** 2012-06-26

**Authors:** Yin Wu, Jie Hu, Eric van Dijk, Marijke C. Leliveld, Xiaolin Zhou

**Affiliations:** 1 Key Laboratory of Child Development and Learning Science, Ministry of Education, Southeast University, Nanjing, China; 2 Center for Brain and Cognitive Sciences and Department of Psychology, Peking University, Beijing, China; 3 Department of Social and Organizational Psychology, Leiden University, Leiden, The Netherlands; 4 Leiden Institute for Brain and Cognition, Leiden University, Leiden, The Netherlands; 5 Department of Marketing, University of Groningen, Groningen, The Netherlands; 6 Key Laboratory of Machine Perception, Ministry of Education, Peking University, Beijing, China; Catholic University of Sacro Cuore, Italy

## Abstract

Previous behavioral studies have shown that initial ownership influences individuals’ fairness consideration and other-regarding behavior. However, it is not entirely clear whether initial ownership influences the brain activity when a recipient evaluates the fairness of asset distribution. In this study, we randomly assigned the bargaining property (monetary reward) to either the allocator or the recipient in the ultimatum game and let participants of the study, acting as recipients, receive either disadvantageous unequal, equal, or advantageous unequal offers from allocators while the event-related potentials (ERPs) were recorded. Behavioral results showed that participants were more likely to reject disadvantageous unequal and equal offers when they initially owned the property as compared to when they did not. The two types of unequal offers evoked more negative going ERPs (the MFN) than the equal offers in an early time window and the differences were not modulated by the initial ownership. In a late time window, however, the P300 responses to division schemes were affected not only by the type of unequal offers but also by whom the property was initially assigned to. These findings suggest that while the MFN may function as a general mechanism that evaluates whether the offer is consistent or inconsistent with the equity rule, the P300 is sensitive to top-down controlled processes, into which factors related to the allocation of attentional resources, including initial ownership and personal interests, come to play.

## Introduction

Individuals tend to value their own possessions more favorably than those they do not own, a bias that has been termed as *mere ownership effect* [1], [2]. This effect occurs even when the actual possessions are imaged, not physically present [3]. The ownership effect has been linked to the self-enhancement motivation in which individuals overvalue an object owned by or associated with self in order to improve their self-image [1].

Recent studies suggested that the perception of ownership modulates other-regarding behavior in economic decision-making [4], [5], [6]. Oxoby and Spraggon [6] asked participants to play a dictator game (DG; [7]) in which the allocator decided how to distribute asset and the recipient had no right but to accept the allocation. The asset (a certain amount of monetary reward) was initially earned either by the allocator or by the recipient through an unrelated task. Result showed that offers to the recipient were lower in the “allocator-earned” condition and higher in the “recipient-earned” condition, highlighting the importance of property right in determining individuals’ other-regarding behavior [8]. Using a related task, Leliveld et al. [4] investigated how the *perception* of ownership affects the allocator’s other-regarding behavior in an ultimatum game (UG). This game, originally developed by Güth et al. [9], is similar to the DG but has one major difference: the recipient can either accept or reject the allocator’s offer. If accepted, the pie is divided as proposed; if rejected, both the allocator and the recipient end empty handed. Leliveld et al. [4] put the chips (related to monetary reward later on) either at the allocator’s side of the table or at the recipient’s side of the table. Results showed that allocations to the recipient were higher in the latter case than in the former case; moreover, this modulation of other-regarding behavior by the perceptions of ownership reflected a true concern for other’s welfare rather than fear of rejection.

The ownership effect is closely related to the concept of *entitlement*. Entitlement is a kind of feeling that may result from ownership: because I feel I own it, I have a right to end up with it. In the above studies, the allocators distributed more assets to themselves when they had the initial ownership of the assets and felt entitled to have more. It should be noted, however, that the previous behavioral studies on the effect of ownership or entitlement have exclusively focused on the allocator’s decision-making behavior. It is not clear how the recipient’s fairness consideration in economic bargain would be affected by the initial ownership or the feeling of entitlement, and more close to the purpose of the present study, whether and how the brain responses to different levels of fairness in asset allocation are modulated by the initial ownership.

This study was therefore conducted to investigate how initial ownership of a bargaining property modulates recipient’s fairness consideration; this was measured through behavioral reactions (i.e., accepting vs. rejecting offers) and electrophysiological recordings. We randomly assigned the property (a certain amount of monetary reward) to either the allocator or the recipient before the presentation of the division scheme and measured the recipient’s event-related potentials (ERPs) evoked by the division scheme. We manipulated the level of fairness in asset allocation by letting the recipient receive disadvantageous unequal offers (1, 2, or 3 out of 10 Chinese yuan), equal offers (5 out of 10 yuan) or advantageous unequal offers (7 or 8 out of 10 yuan). Behaviorally, we were interested in the acceptance rate for different offers. This rate should decrease as the level of fairness in the division scheme decreases. Importantly, this rate could be lower when the assets were initially owned by the recipient than by the allocator, especially when the offers were disadvantageously unequal. The feeling of entitlement might lead the recipient to demand a larger portion of the pie.

Electrophysiologically, we focused on MFN and P300, two ERP components that are sensitive to the evaluation of fairness in asset distribution. The medial frontal negativity (MFN) or the feedback-related negativity (FRN) was originally observed in studies on performance monitoring and the evaluation of decision outcome [10], [11], [12], [13]. The FRN is a negative deflection peaking between 200 ms and 350 ms at frontocentral recording sites, and is more pronounced for negative feedback associated with unfavorable outcomes, such as incorrect responses or monetary loss, than for positive feedback. Later studies showed that these differential responses to decision outcome can be modulated by social factors, such as interpersonal relationship between the evaluator and the decision maker [14], [15], [16], [17], [18], [19], [20] and the extent of personal responsibility for the outcome [21], [22]. These studies suggest the FRN may reflect the processes of assessing the motivational/affective impact of the outcome events (i.e., the processes of putting subjective values onto the outcomes; [10], [23]. Importantly, recent studies extended the role of FRN in outcome evaluation and performance monitoring to the social domain and to the online decision process and found that violations of social norms, such as unfair or unequal offers in asset division, also elicit more negative-going FRN (or MFN) than fair offers [24], [25], [26], [27], [28]. In such studies, participants were offered either fair (e.g., receiving 50%) or unfair (e.g., receiving 10%) divisions of assets (monetary reward) and ERPs were time-locked to the presentation of such division schemes. Although participants were not directly provided with feedback contingent upon their actions or choices, a division scheme may nevertheless be compared with implicit, long-established social norms (e.g., equal division) concerning asset distribution and the scheme’s motivational/affective significance is hence derived. Any violation of the norms would elicit the MFN (FRN) responses. A study showed that individuals highly appreciating moral norms such as fairness and honesty exhibited larger MFN amplitudes when processing unfair offers than individuals with less regard for such norms [24].

For the present design, we predicted a more negative-going MFN for the disadvantageous unequal offers, as compared to the equal offers. The situation for the advantageous unequal offers could be more complex. If the MFN reflects the violation of expectancy at some abstract level, then the advantageous unequal offers should elicit a more negative-going MFN, similar to the disadvantageous unequal offers. If the MFN reflects simply the valence of offers or the relevance of offers to self-interests, then we should predict a less negative-going MFN for the advantageous unequal offers, as compared to the equal offers. Oliveira et al. [29] found that both unexpected positive reward and unexpected negative reward elicited more negative-going FRN responses than expected feedback in an anticipation-timing task and that the two FRN effects were of equal magnitudes, consistent with the first possibility outlined above.

More importantly, we predicted that the initial ownership may modulate the MFN effects for the disadvantageous and advantageous unequal offers. Previous behavioral studies have already shown that people’s fairness consideration is influenced by various social factors, such as the valence (gain vs. loss) of a bargaining property [30], [31], the power of those involved in the transaction [32] and the context in which a division scheme is presented [33], [34]. We also conducted an ERP study asking participants to act as recipients in DG and received either equal or disadvantageous unequal offers from friends or strangers [27]. The MFN effect for disadvantageous unequal offers was present only in the friend-allocation condition, not in the stranger-allocation condition. In the present design, the (dis-)advantageous unequal offers could elicit stronger MFN effects when the bargaining property was initially owned by the recipients rather than by the allocators, as the feeling of entitlement could enhance the recipients’ expectancy towards an equal or larger portion of the pie.

Another ERP component, the P300, which is the most positive peak in the period of 200–600 ms post-onset of feedback and which typically increases in magnitude from frontal to parietal electrodes, has also been found to be related to various aspects of outcome evaluation. Earlier studies employing the oddball paradigm suggested that the P300 is related to higher-order cognitive operations, such as memory updating, selective attention and resource allocation [35]. The P300 has also been found to be related to various aspects of outcome evaluation. Some studies found that the P300 is sensitive to the magnitude of reward, with a more positive response to a larger than to a smaller reward [13], [36]. Other studies suggested that the P300 is also sensitive to reward valence, with a more positive amplitude for positive feedback than for negative outcome [18], [37], [38], [39], [40]. It is possible that during outcome evaluation more attentional resources have been devoted to the outcome magnitude or valence that has stronger motivational significance to the participants. In asset distribution, Wu and colleagues found that the P300 is more positive to fair offers than to disadvantageous unfair offers, suggesting that differential distribution of attentional resources to the two types of offers which had different affective/motivational significance [27]. We hence predicted to observe the same pattern on the P300 for the two types of offers. For advantageous unequal offers, one would normally predict an even more positive P300 as the P300 has been found to increase with the magnitude of reward [13], [36].

It was not clear, however, whether and how the P300 would be modulated by initial ownership. Turk et al. [41] observed a more positive P300 to an object when it was assigned to the participant rather than to another person. The authors interpreted this effect as reflecting increased attention to the self-relevant objects. In the present design, however it was not clear how this initial ownership would affect the P300 responses to the later division schemes.

## Methods

### Participants

Thirty undergraduate and graduate students (11 females) were recruited from the University intranet. The mean age of the participants was 21.6 years, ranging between 19 and 25 years. They were paid 30 Chinese yuan (about $ 4.5) as basic payment and were informed that additional monetary rewards would be paid according to their decisions in the task, although in the end all the participants were paid 20 yuan extra on top of the basic payment. Four graduate students (2 females), who were strangers to the EEG participants, were recruited as confederates. The purpose of using four confederates was to reduce reputation building in the repeated-trial game and to make the experimental setup more realistic since the EEG participant would play against different allocators in rounds of the game.

All the participants were right-handed and had normal or corrected-to-normal vision. They self-reported on a short questionnaire no history of neurological or psychiatric disorders. Informed consent was obtained from each participant before the test. The experiment was carried out in accordance with the Declaration of Helsinki and was approved by the Ethics Committee of the Department of Psychology, Peking University.

### Design and procedures

The experiment had a 3×2 within-participant factorial design, with the first factor referring to the offer type (disadvantageous unequal vs. equal vs. advantageous unequal offer) and the second factor referring to the initial ownership (self vs. other). Disadvantageous unequal offers could be 1, 2, or 3 yuan (out of 10 yuan), equal offers could be 5 yuan (out of 10 yuan), and advantageous unequal offers could be 7 or 8 yuan (out of 10 yuan). The bargaining property (10 yuan) was assigned ostensibly by the computer to either the recipient or the allocator in random order before the division scheme was presented to the recipient.

When the EEG participant came to the laboratory, he/she and the four confederates were told that they would sit in separate rooms to finish a task together through the computer network. The EEG participant was ostensibly selected through lottery to undergo the EEG test. This participant was then told that he/she would play as a recipient in UG and the others would be allocators. He/she was also informed about the rules of UG and the manipulation of ownership. That is, at the beginning of each round the computer would randomly assign 10 yuan to either the allocator or himself/herself, and the allocator would then offer a scheme on how to divide the amount. The EEG participant was asked to press a button with the index finger of his/her left or right hand, without elaborative thinking, to indicate whether he/she would accept or reject the offer. He/she was reminded that the allocators made their division schemes individually and independently, and his/her response would not be sent back to the allocator immediately and therefore could not affect the allocators’ offers in following rounds.

**Figure 1:**
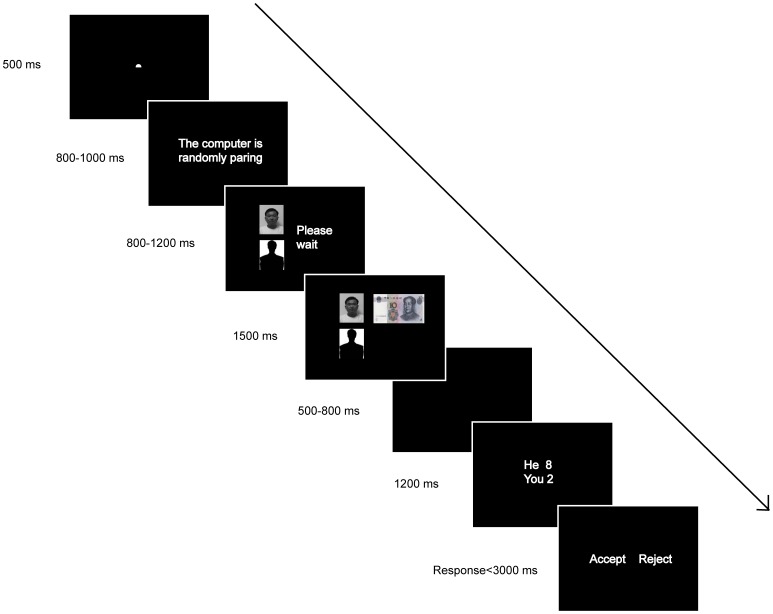
Sequence of events in a single trial.

Each trial began with the presentation of a fixation sign (a white dot subtended 0.3? of visual angle) for 500 ms against a black background (see Fig. 1). The sentence “The computer is randomly pairing” in Chinese (white and Song font, size 32) was presented for either 800, 850, 900, 950, or 1000 ms, indicating to the EEG participant that one of the other four persons was randomly selected to play as an allocator in the current round of game. Then the EEG participant’s own head portrait and a silhouette (each subtended 1.5 × 1.6°, separated for 2.3° between the centers of the two figures) were presented at the left side of the screen for either 800, 900, 1000, 1100 or 1200 ms, along with Chinese words “please wait” (white and Song font, size 32) at the left. This was to suggest to the participant that the computer was assigning the initial ownership of the 10 yuan. The positions of these two figures were counterbalanced over trials. After this frame, the assignment of initial ownership, with a photo of a 10 yuan bill (2.6° × 1.3°) aligned with either of the figures, was presented for 1500 ms. It was explained to the participant that the computer had endowed the money initially to the person involved. After the presentation of a blank screen for a jittered time between 500 and 800 ms, the allocator’s division scheme, in two lines of words (e.g., “he 8, you 2”, white and Song font, size 32) was revealed at the center of screen for 1200 ms. The screen turned blank again for 500 ms, followed by the presentation of two options, “accept” and “reject”, on the left and right side of the screen, with the positions of the two options counterbalanced over participants. The EEG participant was asked to make the “accept” or “reject” decision as quickly as possible and the next trial began after 1000 ms after the button press.

The participant was seated comfortably about 1.5 m in front of a computer screen in a dimly lit room. The experiment was administered on a computer with a Del 22-in. CRT display, using Presentation software (Neurobehavioral System Inc.) to control the presentation and timing of the stimuli. The experiment consisted of 4 blocks of 75 trials each. Under each of the two types of initial ownership, the disadvantageous unequal condition consisted of 20 trials of 1/9 offer, 20 trials of 2/8 offer and 10 trials of 3/7 offer; the equal condition consisted of 40 trials of 5/5 offer; and the advantageous unequal condition consisted of 20 trials of 7/3 offer and 20 trials of 8/2 offer. In addition, 10 trials of 4/6 offer and another 10 trials of 6/4 offer were used as fillers. The number before the slash indicated the offered amount to the recipient and the number after the slash indicated the amount left to the allocator. Without the participant’s knowledge, all the offers were predetermined by a computer program. The 300 trials were pseudo-randomized with the restriction that no more than 3 consecutive trials were of the same offer type and no more than 3 consecutive trials were of the same initial ownership.

A practice block of 20 trials was administered before the formal test. To check the manipulation of initial ownership, we presented the participants, after the experiment, with the fourth frame of Fig. 1. and asked them to indicate on a 7-point Likert Scale to what extent they felt that the property should be in their own possession (1 =  absolutely not in their own possession, 7 =  absolutely in their own possession) and to what extent they felt that the property should be in the allocator’s possession (1 =  absolutely not in the allocator’s possession, 7 =  absolutely in the allocator’s possession) in each initial ownership condition. We also presented the participants with the fourth frame of Fig. 1 and asked the participants to indicate the minimal amount (out of 10 yuan) they wanted from the pie and the fairest division they perceived when the property was assigned to themselves or to the allocator. The participants were debriefed, paid and thanked at the end of the experiment.

### EEG Recording and Analysis

EEGs were recorded from 64 scalp sites using tin electrodes mounted in an elastic cap (Brain Products, Munich, Germany) according to the international 10–20 system. The vertical electrooculogram (VEOGs) was recorded supra-orbitally from the right eye. The horizontal EOG (HEOG) was recorded from electrodes placed at the outer canthus of the left eye. All EEGs and EOGs were referenced online to an external electrode which was placed on the tip of nose and were re-referenced offline to the mean of the left and right mastoids. Electrode impedance was kept below 5 kΩ for EOG channels and for all other electrodes. The bio-signals were amplified with a band-pass from 0.016 to 100 Hz and digitized on-line with a sampling frequency of 500 Hz.

Separate EEG epochs of 1000 ms (with a 200-ms pre-stimulus baseline) were extracted offline, time-locked to the onset of each division scheme. Ocular artifacts were corrected with an eye-movement correction algorithm which employs a regression analysis in combination with artifact averaging [42]. Epochs were baseline-corrected by subtracting from each sample the average activity of that channel during the baseline period. All trails in which EEG voltages exceeded a threshold of ±80 µV during recording were excluded from further analysis. The EEG data were filtered with a band-pass from 0.016 to 30 Hz.

We focused on 10 frontocentral electrodes, FC3, FC1, FCz, FC2, FC4, C3, C1, Cz, C2 and C4 for the MFN responses and 10 centro-posterior electrodes, CP3, CP1, CPz, CP2, CP4, P3, P1, Pz, P2 and P4, for the P300 responses since the MFN and the P300 effects tended to be the strongest on these electrodes. Based on the visual inspection of ERP waveforms, we used the mean amplitudes in the 280–380 ms time window for the MFN measurement and the mean amplitudes in the 400–600 ms time window for the P300 measurement (see also [28] for similar treatment). Average amplitudes over frontocentral and centro-posterior electrodes were used in the following analysis. Analyses of variance (ANOVAs) were conducted with two within-participant factors: initial ownership (self vs. other) and offer type (disadvantageous unequal vs. equal vs. advantageous unequal offer). The Greenhouse-Geisser correction for violation of the assumption of sphericity was applied where appropriate. The Bonferroni correction was used for multiple comparisons.

## Results

Among the thirty EEG participants, three participants stated that they completely disbelieved the setup of the experiment in the interview after the EEG test, four participants displayed excessive artifacts in EEG recording, one participant misunderstood the game rule, and one participant accepted all the offer types. These participants were excluded from data analysis, leaving twenty-one participants (8 females) for the following analysis.

### Manipulation Checks of Initial Ownership

The post-experiment questionnaire indicated that the incidental assignment of the 10 yuan bill in line with either the participant’s head portrait or the other’s silhouette strongly affected the participants’ perception of potential ownership. A 2 (location of the 10 yuan bill: the recipient’s head portrait vs. the other’s silhouette) × 2 (benefactor of allocation: allocator vs. recipient) repeated measures ANOVA on the perceived ownership showed a significant interaction between the two factors, *F*(1, 20) = 56.88, *p*<0.001. Simple-effect tests revealed that when the 10 yuan bill was temporarily located in line with the participant’s own portrait, participants thought that the property should be more in their own possession (mean ± SE, 5.24±0.28) than in the allocator’s possession (2.90±0.25), *p*<0.001. On the other hand, the participant perceived the property to be more in the allocator’s possession (5.14±0.29) than in their own (3.14±0.32), *p*<0.01, when the 10 yuan bill was located in line with the other’s silhouette.

The manipulation of initial ownership also influenced the participants’ self-reported minimal acceptance amount out of 10 yuan. The minimal acceptance amount was significantly higher when the property was initially endowed to the participant (4.86±0.33) than when the bill was initially endowed to the allocator (2.86±0.33), *p*<0.001. Moreover, the participants indicated that the fairest offer for themselves was 6.48±0.25 yuan (out of 10 yuan) when the property was initially endowed to the participant, which was significantly higher than the amount when the bill was initially endowed to the allocator (4.67±0.26), *p*<0.001. These results indicate that the perceived fairness in asset allocation changes according to the initial ownership or the feeling of entitlement.

### Behavioral Results

**Figure 2:**
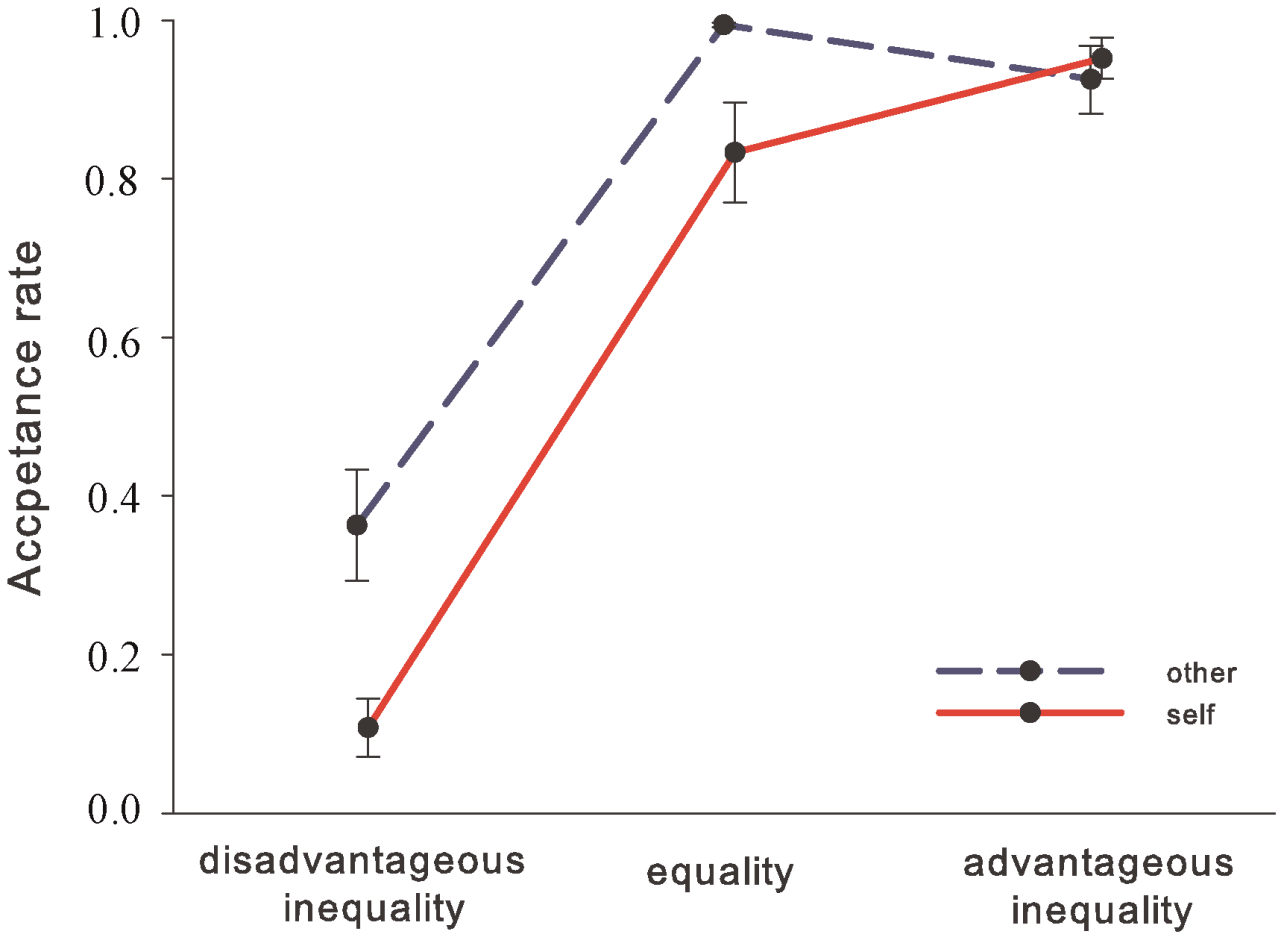
The acceptance rate in the ultimatum game as a function of the offer type. Error bars represent standard errors of the means.

The acceptance rates for different division schemes are presented in Fig. 2. A 2 (initial ownership: self vs. other) × 3 (offer type: disadvantageous unequal vs. equal vs. advantageous unequal offer) repeated measures ANOVA revealed a significant main effect of offer type, *F*(2, 40) = 108.31, *p*<0.001, indicating that the acceptance rate for disadvantageous unequal offers (0.24±0.05) was lower than for either equal (0.91±0.03) or advantageous unequal offers (0.94±0.03), as confirmed by the post-hoc tests (*p*<0.001). The differences between the equal and the advantageous unequal offer conditions were not significant (*p*>0.1). The main effect of initial ownership was also significant, *F*(1, 20) = 12.24, *p*<0.01, suggesting that the acceptance rate was higher when the 10 yuan bill was initially aligned with the other’s silhouette (0.76±0.02) than when bill was presented with the participant’s own portrait (0.63±0.03). Importantly, the interaction between initial ownership and offer type was significant, *F*(2, 40) = 7.25, *p*<0.01. Simple-effect tests showed that the acceptance rate to disadvantageous unequal offers was significantly higher in the “other” condition (0.36±0.07) than in the “self” condition (0.11±0.04), *t*(20) = 3.81, *p*<0.01. A similar pattern was observed for equal offers (0.99±0.003 vs. 0.83±0.06), *t*(20) = 2.59, *p*<0.05. However, this effect was absent for advantageous unequal offers, *t*(20) = –0.70, *p*>0.1.

### ERP Responses to the Presentation of Division Schemes

**Figure 3:**
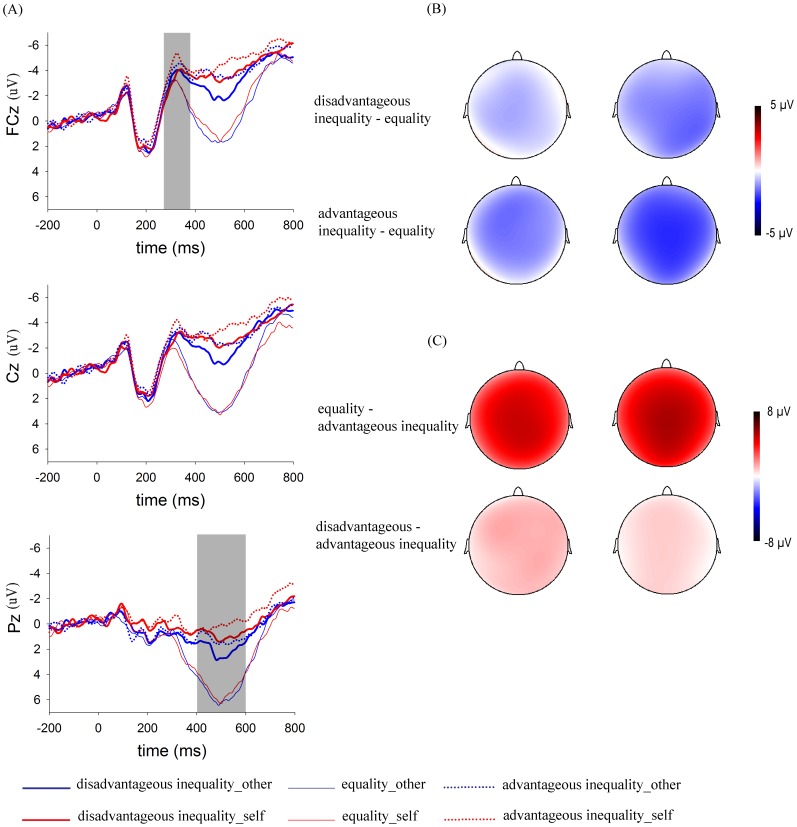
ERP responses and topographic maps. (**A**) ERP responses time-locked to the onset of different offers at the midline FCz, Cz and Pz. The shaded 280–380 ms time window was for the calculation of the mean amplitudes of the MFN. The shaded 400–600 ms time window was for the calculation of the mean amplitudes of the P300. (**B**) Topographic maps for the MFN effects in the 280–380 ms time window. (**C**) Topographic maps for the P300 effects in the 400–600 ms time window.

For the mean amplitudes in the 280–380 ms (MFN) time window (Fig. 3A and 3B), a 2 (initial ownership: self vs. other) × 3 (offer type: disadvantageous unequal vs. equal vs. advantageous unequal offer) repeated-measures ANOVA showed a significant main effect of offer type, *F*(2, 40) = 8.66, *p*<0.01, indicating that ERP responses were more negative-going for disadvantageous (−2.06 µV) and advantageous unequal offers (−2.72 µV) than for equal offers (−1.19 µV), *p* = 0.06 and *p*<0.01, respectively. The ERP responses to the two types of unequal offers did not differ, *p*>0.1. However, we found no significant main effect of initial ownership, *F*(1, 20) = 0.18, *p*>0.1, nor the interaction between initial ownership and offer type, *F*(2, 40) = 1.13, *p*>0.1.

Similarly, for the mean amplitudes in the 400–600 ms (P300) time window (Fig. 3A and 3C), the 2 × 3 ANOVA showed also a main effect of offer type, *F*(2, 40) = 32.98, *p*<0.001, indicating that the mean amplitudes were more positive for equal offers (4.36 µV) than for disadvantageous unequal offers (0.94 µV) or advantageous unequal offers (0.12 µV). The differences between conditions were all significant after Bonferroni correction, *p*<0.001 or *p*<0.01. The main effect of initial ownership was also significant, *F*(1, 20) = 8.28, *p*<0.01, suggesting that the ERP responses were more positive for the “other” (2.12 µV) than for the “self” condition (1.49 µV). The interaction between offer type and initial ownership did not reach significance, *F*(2,40) = 0.54, *p*>0.1, indicating that the initial ownership effect on the P300 was essentially the same across the three types of offers.

## Discussion

This study demonstrated that initial ownership influenced recipients’ brain responses to unfair asset allocation schemes and their behavioral decisions to accept offers. Participants were more reluctant to accept disadvantageous unequal and equal offers when the bargaining property was initially in their own possession than when it was in the other’s possession, and this distinction disappeared for advantageous unequal offers. Electrophysiologically, both disadvantageous and advantageous unequal offers elicited more negative going ERP responses compared to equal offers in an earlier, MFN time window (280–380 ms), with no obvious differences between the two types of unequal offers. These earlier effects were not affected by the initial ownership. In a later time window (400–600 ms), however, the P300 was more positive for equal offers than for disadvantageous unequal or advantageous unequal offers and were more positive when the bargaining property was initially owned by the allocator than by the recipient. In the following paragraphs, we explore the implications of our behavioral and electrophysiological findings, focusing on the effects of fairness in asset allocation and the effects of initial ownership.

Previous studies have shown that noncausal forms of association between an individual and an object (e.g., the numbers corresponding to an individual’s birthday, prior touch or use of the object) can significantly increase the individual’s preference or valuation of the object [43], [44], [45], [46]. In this study, the initial random assignment of the 10 yuan bill strongly affected the participants’ perception of ownership and the feeling of entitlement in subsequent asset distribution, as demonstrated by their post-experiment self-report. Although the participants had been explicitly told that the initial assignment was randomly conducted by computer and it did not imply that they would eventually have the money, this perception of ownership and feeling of entitlement had nevertheless affected the participants’ subsequent acceptance or rejection of disadvantageously unfair and even fair (equal) offers.

One of the prominent motivations for individuals rejecting disadvantageous unequal offers in asset distribution is to preserve self-image/self-esteem and/or to punish unfair behavior [47], [48]. The assignment of initial ownership to a participant may increase his feeling of entitlement, and disadvantageous unequal divisions and even equal divisions would be perceived as challenges to his/her self-image or self-esteem. These challenges would then meet strong reactions, resulting in lower acceptance rates. Thus, the perceived fairness or equity in asset distribution can be highly context-dependent [33], [34].

On the other hand, the high acceptance rate and the absence of initial ownership effect for advantageous unequal offers can also be taken as evidence for the context-dependent nature of fairness consideration. Although individuals care for fairness in asset distribution, particularly when they are in a disadvantageous position [30], [31], they are nevertheless self-interested. This care for self-interests may be strategic and is shown when their self-interests are not likely to be negated. In this situation, effects of other social factors (including the initial ownership) are dwarfed or overshadowed.

The finding of an MFN effect, with more negative going responses to disadvantageous unequal offers than to equal offers, replicated previous studies [24], [25], [26], [27], [28]. This effect may reflect the detection of social expectancy violation as egalitarian distribution of assets is an expected social norm in our life [49], [50], [51]. The human brain might have developed specific mechanisms to detect ongoing deviation from social norms [52] and these mechanisms might be based on similar neural substrates as those engaged in detecting errors during non-social reinforcement leaning [53]. For instance, a recent fMRI study on social conformity in facial attractiveness judgment showed that conflict with group opinions, regardless of whether the opinions were given by human peers or by computers, triggered activation of brain regions implicated in reinforcement learning, i.e., rostral cingulate zone and the ventral striatum, and these neural signals can predict whether the participants would subsequently change their initial judgment [54].

Importantly, we found that the advantageous unequal offers also elicited more negative-going MFN responses than equal offers, and this effect appeared to be of equal magnitude as for disadvantageous unequal offers. This finding is novel and important because it allows us to differentiate theoretical proposals concerning the nature of MFN or FRN (assuming they are essential the same, as we argued in the Introduction). One proposal is that the FRN reflects the impact of midbrain dopamine signals on the anterior cingulate cortex (ACC) [11], [55]. The phasic decreases in dopamine inputs elicited by negative prediction errors (i.e., “the result is worse than expected”) give rise to the increased ACC (anterior cingulate cortex) activity that is reflected as larger MFN amplitude, whereas the phasic increases in dopamine signals elicited by positive prediction errors (i.e., “the result is better than expected”) give rise to decreased ACC activity that is reflected as smaller MFN amplitudes. By this account, we should expect to observe *less negative* (or *more positive*) *going* MFN responses to advantageous unequal offers than to equal offers. Another more viable conception is that the FRN (the associated ACC) serves as a general performance monitoring system which detects violation of (social and non-social) expectancy, irrespective of whether the violated expectancy is positive or negative [29], [39], [56]. By this account, although the advantageous unequal offers could benefit the recipients (i.e., better than expected), they nevertheless violated the equity rule in asset distribution [57], [58], just as the disadvantageous unequal offers. Thus any division schemes in violation of the equity rule would be detected by the monitoring system, resulting in more negative-going MFN responses (see also [29], [39], [56]). Indeed, recent studies suggest that the short-latency phasic responses in the dopamine system are related to a general process of switching attention to unexpected, behaviorally relevant stimuli [59]. The feedback, whether positive or negative, elicits a phasic increase in the activity of mesencephalic dopamine neurons which, in turn, induces increased excitability in the ACC, thereby giving rise to the FRN/MFN effect.

A perhaps surprising finding in this study was that the initial ownership of the distributed asset had no obvious effect on the MFN responses to division schemes. This absence of an initial ownership effect appears to be at odds with Wu et al. [27] in which the social distance between the recipient and the allocator (being a friend or a stranger) modulated the MFN responses to fair and disadvantageous unfair offers. We believe that the discrepancy between the two studies may be related to paradigms adopted for the experiments. Wu et al. [27] used a DG task in which the recipient had no choice but to accept any offers given by the allocator. However, in the UG task used here, the recipient could choose either to accept or to reject offers. Thus the outcome to the recipient was deterministic in DG and was negotiable in UG. This difference in the certainty of outcome may affect the level of affective/motivation significance assessment for the offers, as deeper or more comprehensive assessment in DG allows social/affective factors to play a bigger role. In UG, however, the system may adopt a “wait-and-see” strategy and conduct deeper assessment of offers only at a later stage involving more top-down processes [18], [19], [60], [61], rendering a P300 effect for the initial ownership (see later discussion).

Note that the assignment of bargaining property to the participant may enhance their demand for a larger portion of the pie, evidenced by the reduced acceptance rate for the equal division in the “self” condition than in the “other” condition. One might view this enhanced demand for the pie in self-ownership as a kind of social norm. Consequently if the MFN reflects the violation of social norm, we should expect to observe more negative-going MFN responses to the disadvantageous offers in the “self” condition than in the “other” condition. Although we did obtained numerically larger MFN responses for the former (−2.21 µV) than for the latter (−1.92 µV), the difference between the two conditions did not reach statistical significance. It is possible that when different social norms are involved in evaluating schemes of asset division, the equity rule, which is ubiquitous in the society, might dominate over other rules, including the rule for a larger portion of pie in self-ownership, in determining the MFN responses. Further studies are needed to investigate how different social norms or rules might interact to modulate the brain activity in outcome evaluation or interpersonal interaction.

In contrast to the MFN, we found that the P300 was modulated by both the offer type and the initial ownership, although these modulations were independent from each other. Previous studies on outcome evaluation have indicated that the P300 is related to processes of attentional allocation [62], [63] and/or high-level motivational/affective evaluation [13], [64]. According to the equity theory [57], [58], individuals who are facing inequity would feel distressed and are less satisfied with asset distribution than individuals who are facing equity. The stronger P300 responses to equal offers than to unequal offers may suggest that participants (recipients of asset distribution) in this study attached more motivational/affective significance to the equal divisions than to unequal divisions, consistent with the social fairness norms cultured in individuals.

In addition, we found that disadvantageous unequal offers elicited more positive P300 than advantageous unequal offers. Although both types of offers violate the equity rule of social norms, it is possible that different amount of attentional resources are used to process the two types of offers. For disadvantageous unequal offers, participants might be in a difficult position to assess the pros and cons of accepting or rejecting offers; for advantageous unequal offers, participants might not have such dilemma and they can assess the implications of offers, as demonstrated by their 94% acceptance rate.

On the other hand, we observed a small, but significant initial ownership effect on the P300, with the offers from the bargaining property initially owned by allocator eliciting more positive P300 responses than the offers from the property initially owned by the recipient himself/herself. A number of studies on outcome evaluation have shown that the P300 is sensitive to reward valence in gambling tasks, with positive outcomes eliciting more positive P300 than negative outcomes [18], [37], [38], [39], [40]. In the present study, any amount proposed by the allocator in the “other” condition might be considered, implicitly, as a kind of extra “gain”, even though the recipient may eventually decide to reject the offer and lose it. Conversely, any amount proposed by the allocator in the “self” condition might be considered as a kind of “loss” as the bargaining property was initially assigned to the recipient and he/she might implicitly declare the ownership of the whole lot (see also [65]).

An important finding here was that the modulations of the P300 by offer type and initial ownership appeared to be independent from each other, consistent with the absence of an interaction between fairness of offers and social distance between the allocator and recipient in DG [27]. We would like to suggest that there are two top-down processes associated with the P300. One process cares for fairness of different offers, with different levels of attentional resources being devoted to the elaborative processing of the social/affective significance of offers. Another process cares more for self-interests and is sensitive to gain/loss. Either of two processes can modulate the P300 magnitude, although it needs further investigation to elucidate under what circumstances the two processes work independently when they are manipulated concurrently.

In summary, by assigning a bargaining property to either the allocator or the recipient and presenting the recipient with offers of different fairness levels, we found that the participant, acting as the recipient, were more likely to reject disadvantageous unequal and equal offers when they initially owned the property than when they did not. The two types of unequal offers evoked more negative-going MFN than the equal offers in an early time window (280–380 ms) and these differential effects were not modulated by the initial ownership. In a late time window (400–600 ms), however, the P300 responses to division schemes were affected not only by offer types but also by whom the property was initially assigned to. These findings suggest that while the MFN may function as a general mechanism that evaluates whether the offer is consistent or inconsistent with the equity rule, the P300 is sensitive to later, top-down controlled processes, into which factors related to the allocation of attentional resources, including initial ownership and personal interests, come to play.
